# Skin and mucosal manifestations of an AIDS-related systemic mycosis

**DOI:** 10.4102/sajhivmed.v22i1.1198

**Published:** 2021-01-28

**Authors:** Michael T. Boswell, Liam Robinson, Nelesh Govender

**Affiliations:** 1 Department of Internal Medicine, School of Medicine, Faculty of Health Sciences, University of Pretoria, Pretoria, South Africa; 2 Department of Internal Medicine, Steve Biko Academic Hospital, Pretoria, South Africa; 3 Department of Oral Pathology and Oral Biology, School of Dentistry, Faculty of Health Sciences, University of Pretoria, Pretoria, South Africa; 4 Centre for HAIs, AMR and Mycoses, National Institute for Communicable Diseases, Johannesburg, South Africa; 5 Faculty of Health Sciences, University of the Witwatersrand, Johannesburg, South Africa

**FIGURE 1:**
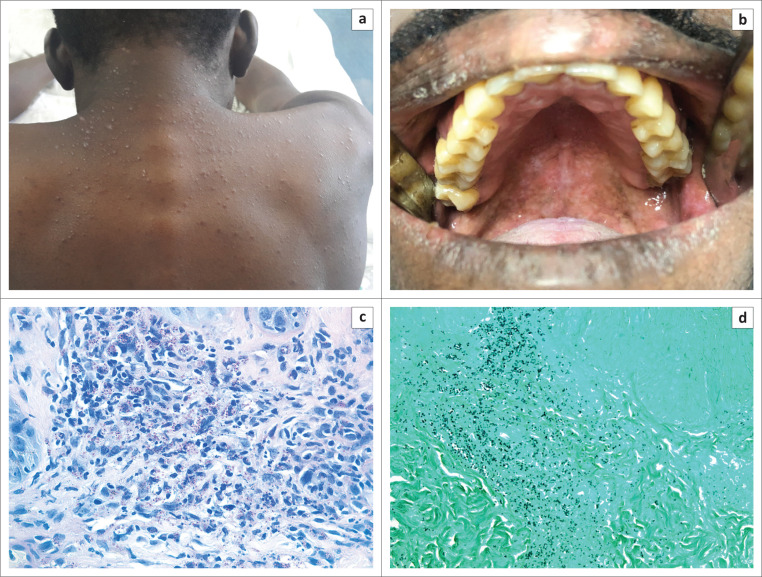
(a) Erythematous to flesh-coloured papules on the trunk; (b) Granular-appearing, pigmented lesions involving the hard and soft palate; (c) PAS-D and (d) Grocott-Gomori stained sections highlighting the intracytoplasmic organisms (original magnification × 200).

A human immunodeficiency virus (HIV)-positive male from Cameroon who had recently started antiretroviral therapy presented with a new rash, night sweats and loss of weight. On examination, erythematous to flesh-coloured papules were noted on the trunk (a). Intraoral examination revealed granular-appearing lesions of the hard and soft palate, with areas of pigmentation in keeping with HIV-associated mucosal hyperpigmentation (b). A full blood count showed a pancytopenia, with a moderate neutropenia. He had a severe lymphopenia, and his CD4+ T-cell count was 46 cells/microlitre (µL). Serum (1-3)-β-d-glucan and ferritin levels were markedly elevated at > 500 picograms per millilitre (pg/mL) and 5533 micrograms per litre (µg/L), respectively. Periodic Acid–Schiff with Diastase (PAS-D) and Grocott-Gomori histochemical stains of a skin punch biopsy showed numerous small, round intracytoplasmic organisms within histiocytes, consistent with histoplasmosis (c and d) (see Figure 1). A pan-fungal polymerase chain reaction (PCR) assay confirmed infection with either *Histoplasma capsulatum* or *Emergomyces africanus*. This PCR assay cross-reacts with *Blastomyces* species; however, the yeast phase of this pathogen has a different histological appearance.

